# Personalized medicine in psoriasis: developing a genomic classifier to predict histological response to Alefacept

**DOI:** 10.1186/1471-5945-10-1

**Published:** 2010-02-12

**Authors:** Mayte Suárez-Fariñas, Kejal R Shah, Asifa S Haider, James G Krueger, Michelle A Lowes

**Affiliations:** 1 Laboratory for Investigative Dermatology, The Rockefeller University 1230 York Ave, New York, NY, 10065, USA; 2 Center for Clinical and Translational Science, The Rockefeller University, 1230 York Avenue, New York, NY, 10065, USA

## Abstract

**Background:**

Alefacept treatment is highly effective in a select group patients with moderate-to-severe psoriasis, and is an ideal candidate to develop systems to predict who will respond to therapy. A clinical trial of 22 patients with moderate to severe psoriasis treated with alefacept was conducted in 2002-2003, as a mechanism of action study. Patients were classified as responders or non-responders to alefacept based on histological criteria. Results of the original mechanism of action study have been published. Peripheral blood was collected at the start of this clinical trial, and a prior analysis demonstrated that gene expression in PBMCs differed between responders and non-responders, however, the analysis performed could not be used to predict response.

**Methods:**

Microarray data from PBMCs of 16 of these patients was analyzed to generate a treatment response classifier. We used a discriminant analysis method that performs sample classification from gene expression data, via "nearest shrunken centroid method". Centroids are the average gene expression for each gene in each class divided by the within-class standard deviation for that gene.

**Results:**

A disease response classifier using 23 genes was created to accurately predict response to alefacept (12.3% error rate). While the genes in this classifier should be considered as a group, some of the individual genes are of great interest, for example, cAMP response element modulator (CREM), v-MAF avian musculoaponeurotic fibrosarcoma oncogene family (MAFF), chloride intracellular channel protein 1 (CLIC1, also called NCC27), NLR family, pyrin domain-containing 1 (NLRP1), and CCL5 (chemokine, cc motif, ligand 5, also called regulated upon activation, normally T expressed, and presumably secreted/RANTES).

**Conclusions:**

Although this study is small, and based on analysis of existing microarray data, we demonstrate that a treatment response classifier for alefacept can be created using gene expression of PBMCs in psoriasis. This preliminary study may provide a useful tool to predict response of psoriatic patients to alefacept.

## Background

Developing biomarkers that predict response to therapy is an ambitious goal of modern medicine. This is an aspect of personalized medicine that could transform our ability to treat patients successfully with a particular therapy in a cost-effective manner. Alefacept, an anti-CD2 fusion protein (Amevive, Astellas Pharma), is a biologic agent that often induces a remarkably durable remission [1]. However, it produces a *PASI *75 response (Psoriasis Area and Severity Index [PASI] response of greater than 75% improvement from baseline) in only approximately 30-50% of patients. Thus alefacept is an excellent example of a treatment that would benefit from being able to predict which patients with psoriasis would respond to this agent, and which patients might not respond.

The results of our original mechanism of action study of alefacept have already been published [2,3]. In brief, patients were classified as histologic responders or non-responders, as described in the Methods section. Patients that responded to alefacept showed reductions in tissue gene expression of IFNγ, signal transducer and activator of transcription 1 (STAT-1), monokine induced by IFNγ (MIG), inducible NO synthase (iNOS), IL-8, and IL-23, as well as myeloid DCs (measured by immunohistochemistry for CD11c^+ ^and CD83^+ ^cells). As alefacept bound primarily to T cells and not DCs, we suggested that T cells were the primary target for therapy, but that DCs and a spectrum of type 1 inflammatory genes were coordinately suppressed. Furthermore, we demonstrated by FACS of PBMCs that in all patients, alefacept treatment caused a preferential decrease in effector memory T cells (CCR7^- ^CD45RA^-^) for both CD4^+ ^and CD8^+ ^T effector memory cells. In contrast, central memory T cells (CCR7^+^CD45RA^-^) were less affected, and naïve T cells (CCR7^+^CD45RA^+^) were relatively spared. Circulating CD8^+ ^effector T cells and Type 1 T cells (IFN-γ-producing) were also significantly reduced [2,3].

The primary mechanism of action of alefacept is considered to be by killing CD2^+ ^T cells by a cytotoxic mechanism (involving NK cell bridging), or by blocking CD2 signaling [4,5]. In a previous study [6], our group established a new therapeutic mechanism for alefacept in psoriasis, as it also serves as an agonist for CD2 and induces positive T cell signaling responses. In this study, we analyzed genomic expression of circulating PBMCs, comparing baseline versus 24 hour time-point. During the first day of treatment in PBMCs, there was suppression of inflammatory genes, but perhaps surprisingly, a marked induction of mRNAs for STAT1, IL-8, and MIG. These agonistic effects of alefacept in PBMC were confirmed *in vitro*. These data demonstrated that alefacept activates gene expression in leukocytes and suggested that its therapeutic action may be as a mixed agonist/antagonist.

These findings suggested that differential activation of genes may categorize clinical responders to alefacept, and gave the first indication of differences in the pre-treatment circulating leukocytes in responders and non-responders. Thus these results led us to ask whether baseline gene expression in PBMCs might be used to classify responders versus non-responders and predict a priori who would respond to alefacept. This would have a dual benefit, allowing those responders to receive treatment with confidence, and sparing those who would not respond the cost, potential serious immunosuppressive effects and inconvenience of a course of therapy. The aim of this study was to mine our existing genomic data using alternative, previously developed analytic methods to generate a "genomic classifier" [7], a set of genes that could specifically predict response to alefacept. This "genomic classifier" could then be tested in a prospective clinical trial of alefacept in psoriasis.

Genomic expression profiles have been successfully used for disease classification and to predict response to treatment. In a seminal paper in 1999, Golub *et al *demonstrated that the type of haematological malignancy could be determined by class prediction using microarray data [8]. Since then, other investigators have shown that genomic patterns of expression could be used to predict the progression and prognosis of cancer [9]. Gene expression profiling of neoplastic tissue has been performed to develop a genomic classifier for response to a chemotherapy regimen for patients with advance colorectal cancer [10], or doxorubicin sensitivity in gastric cancers [11]. Genomic classifiers have also been developed in breast cancer to predict tamoxifen-resistance [12], and docetaxel response [13]. In chronic inflammation such as rheumatoid arthritis, response to etanercept (Enbrel, TNF-inhibitor) could be predicted by a genomic classifier consisting of specific combinations of gene doublets and triplets [14].

## Methods

### Clinical trial

An IRB-approved clinical trial was conducted at Rockefeller University in 2002-2003, treating 22 patients with moderate-to-severe psoriasis with alefacept (7.5-mg weekly i.v. ×12 weeks). The initial aim of the clinical trial was to conduct a mechanism of action study, and the study was powered to produce groups of at least six patients that could be designated as responders versus non-responders (as defined below) to alefacept. Patients were recruited from local dermatologists, and by IRB-approved radio and print advertisements. 19 males and 3 females, (ages 29-68 years, median 49 years) were enrolled. Major inclusion criteria were: involvement of psoriasis vulgaris of >10% body surface area, no systemic treatment for at least 4 weeks before entering the study, no significant infections or immunosuppression, and no significant renal, hepatic, or other medical disease. Informed consent was obtained. The results of tissue analysis and peripheral blood analysis describing the mechanism of action of this biologic agent have already been published [2,3], and are discussed in the Background section.

**Figure 1:**
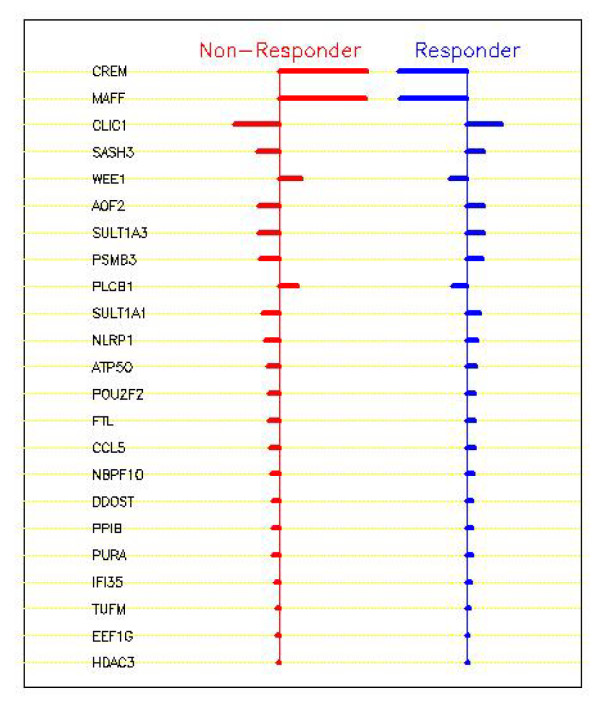
**Histological classification of response**. Clinical photographs, haematoxylin and eosin (H&E) and keratin 16 staining of non-lesional skin, and lesional skin pre and post alefacept treatment in (A) a responding patient and (B) a non-responding patient. Lesional skin in both patients demonstrated characteristic features of psoriasis: epidermal acanthosis, parakeratosis, loss of the granular layer, elongation of the rete, dilated blood vessels, and a dense inflammatory infiltrate in the dermis. There was strong K16 staining. Only the responding patient showed resolution of inflammation and K16 staining to non-lesional appearance.

Tissue samples were collected before and during the trial at baseline (non-lesional and lesional), week 2, week 6 and week 13. The patients were categorized as responders or non-responders based on histological changes in their skin biopsies over the course of the clinical trial (Figure 1) [2]. Histological response of psoriatic lesions was defined as normalization of keratin 16 (K16) expression, reduction of epidermal hyperplasia, restoration of a granular layer, and orthokeratosis in week-13 biopsies. Overall, 22 patients were enrolled, 2 dropped out due to non-response. 12 patients were classified as responders, and 10 as non-responders (8 patients were categorized as non-responders based on histological analysis, 10 patients were non-responders based on intent-to-treat). High quality microarray data were available on 9 responders and 7 non-responders.

### Processing of specimens for microarray

Initial microarray data from PBMCs of these patients has been published [6]. Briefly, peripheral blood draws were taken before alefacept administration. PBMCs were isolated and stored at -80°C, until required. Pre-treatment RNA was extracted, and hybridized to HGU95Av2 Affymetrix Gene Chip containing probe sets representing 12,000 genes, using standard methods.

### Quality Control, Pre-processing and Filtering

Gene Chip CEL files were scrutinized for spatial artefacts using Harshlight package https://mustat.rockefeller.edu/harshlight[15]. Intensity values (CEL files) were pre-processed to obtained expression values using GCRMA algorithm. Expression values were filtered to eliminate probe sets with low variation or low intensity. Probe sets with standard deviation greater than 0.3, and expression values greater than 3 in at least 1 sample, were kept for further analysis, leaving a total of 5218 probe sets. Genes were annotated using up-to date annotation from of HGU95av2 chips available at Bioconductor. The data discussed in this publication have been deposited in NCBI's Gene Expression Omnibus and are accessible through GEO Series accession number (GSE18948).

### Discriminant Analysis

We used a discriminant analysis method that performs sample classification from gene expression data, via "nearest shrunken centroid method" [7]. This method is a modification of the conventional nearest centroid method [16], where centroids for each gene (average gene expression for the gene in each class) are divided by the within-class standard deviation in order to give more weight to genes with smaller variations across samples in the same class. Then, for prediction, every new sample is classified based on the gene expression profile of the sample by the following prediction rule: The class whose centroid is closest (by euclidean distance) to the gene expression profile of the sample is the predicted class for that new sample.

Nearest shrunken centroid classification makes one important modification to conventional nearest centroid classification. It "shrinks" each of the class centroids toward the overall centroid for all classes by an amount called the "threshold". This shrinkage consists of moving the centroid towards zero by threshold, setting it equal to zero if it hits zero: i.e if threshold = 2, a centroid of 3.2 would be shrunk to 1.2 and a centroid of 1.2 would be shrunk to zero. After shrinking the centroids, the new sample is classified by the prediction rule of the nearest centroid method (see above), but using the shrunken class centroids. This method has two advantages: it can make the classifier more accurate by reducing the effect of noisy genes, and it performs automatic gene selection. If a gene is shrunk to zero for all classes, then it is eliminated from the classifier. Alternatively, it may be set to zero for all classes except one, indicating that high or low expression for that gene characterizes that class.

**Figure 2:**
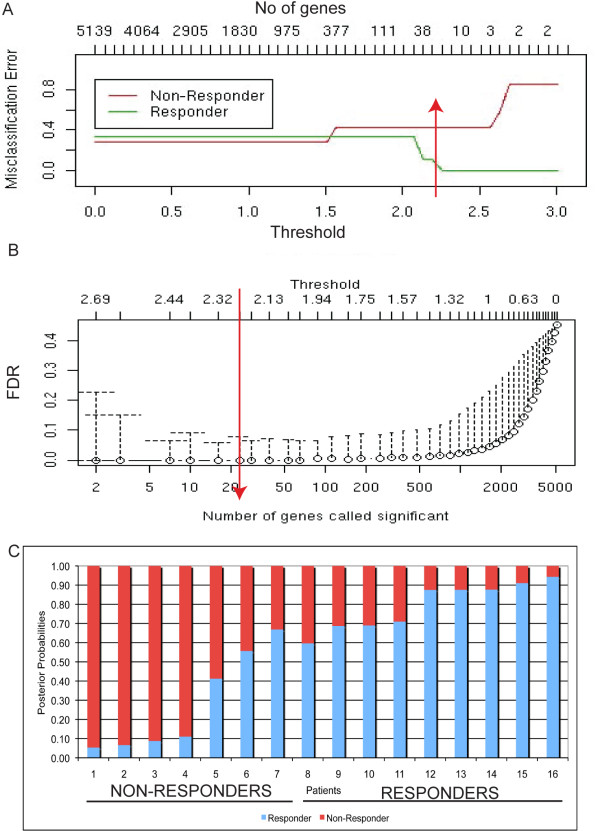
**Determining the genomic classifier by discriminant analysis**. (A) The misclassification error (y-axis) as a function of the threshold (x-axis) and the number of genes (top x-axis). The red arrow identifies the optimal threshold (2.25), which gave an overall error rate of 0.123. (B) Median and 90th percentile of the false discovery rate (FDR, y-axis) as a function of threshold (top x-axis), and number of genes (x-axis). The red arrow identifies the optimal threshold (2.25). (C) The posterior probability of the patients in our trial being classified as a non-responder (<0.5, red) or a responder (>0.5, blue). Patients 1-7 were histological non-responders, and patients 8-16 were responders. Two patients 6 and 7 were mis-identified by the classifier as responders when they were histological non-responders.

The value of the threshold (which determines the number of genes in the final classifier) is set by the user based on the predictive performance of the classifier. In this study, we used 7-fold cross validation to assess the misclassification error (Figure 2A) and the resulting false discovery rate (FDR) for the genes in the classifier (Figure 2B). The value of the threshold was set to the value that minimized both statistics (Figure 2A and 2B). Once the classifier is built (by establishing the genes involved the prediction rule), the probability of being classified in each class can be calculated for each sample/patient (Figure 2C). The package pamr from R http://www.R-project.org/ was used to perform this analysis. There is insufficient RNA remaining from this trial to conduct confirmative PCR experiments.

## Results and Discussion

The age, gender and ethnicity of the patients, as well as PASI scores during the trial are described in Table 1. An example of the clinical photography, histology and K16 immunohistochemistry for a responding (Figure 1A) and a non-responding patient (Figure 1B) are demonstrated. Lesional skin of responding patients showed epidermal acanthosis, parakeratosis, loss of the granular layer, elongation of the rete, dilated blood vessels, and a dense inflammatory infiltrate in the dermis. There was strong K16 staining throughout the epidermis. In responding patients, at the end of treatment there was resolution of cutaneous inflammation almost to the appearance of non-lesional skin. This was quite different in the non-responding patients, as the histology of the psoriasis plaque and K16 immuno-staining at the end of treatment were similar to lesional skin the beginning of the trial.

**Table 1:** Demographic details and PASI scores for patients used to develop genomic classifier to alefacept.

Patients	Age	Gender	Ethnicity	Response	PASI Baseline	PASI End of treatment
1	33	M	Caucasian	NR	6.1	4.3

2	52	M	Caucasian	NR	6.8	5.3

3	49	M	Hispanic	NR	23.0	19.4

4	59	M	Caucasian	NR	43.2	32.2

5	59	M	Caucasian	NR	17.7	*NA

6	55	M	Caucasian	NR	34.3	22.8

7	38	M	Caucasian	NR	9.3	4.7

8	49	M	Caucasian	R	10.9	2.7

9	43	M	Caucasian	R	26.1	19.3

10	45	M	African American	R	17.1	2.2

11	36	M	Asian	R	17.1	2.3

12	44	M	Hispanic	R	35.0	5.2

13	41	M	Caucasian	R	20.7	5.9

14	68	M	Caucasian	R	17.1	14.4

15	62	M	Caucasian	R	9.4	3.8

16	29	F	Hispanic	R	16.8	2.0

* Little clinical improvement after 10 weeks of treatment; PASI difficult to determine due to skin infection.

Microarray data on 9 responders and 7 non-responders were available for further analysis using the discriminant analysis method. Figure 2 shows the misclassification error rate (Figure 2A) and the false discovery rate (Figure 2B) for thresholds ranging from 0-3. A threshold of 2.25 was chosen to create the final predictor (red arrow), as it rendered the best performance of the classifier in the cross-validation stage. The error rate for this final predictor was 0.123 (12.3%), as two non-responder patients were incorrectly classified as responders (patients 6 and 7, Figure 2C). However, the classifier correctly identified all responders as responders (patients 8-16). Classifying a responder as a non-responder would be the most costly error in terms of patient treatment, because otherwise a potential responder patient would be incorrectly left without treatment. However, this is still an improvement over the current situation as clinicians try to decide which therapeutic agent is best for a given patient. Approximately four out of ten patients would respond to alefacept (given a 30-50% therapeutic response rate); if this predictor is validated with the same error rate (12.3%), approximately five out of six patients would respond to alefacept. The false discovery rate for this final predictor was smaller than 0.1 (Figure 2B).

**Figure 3:**
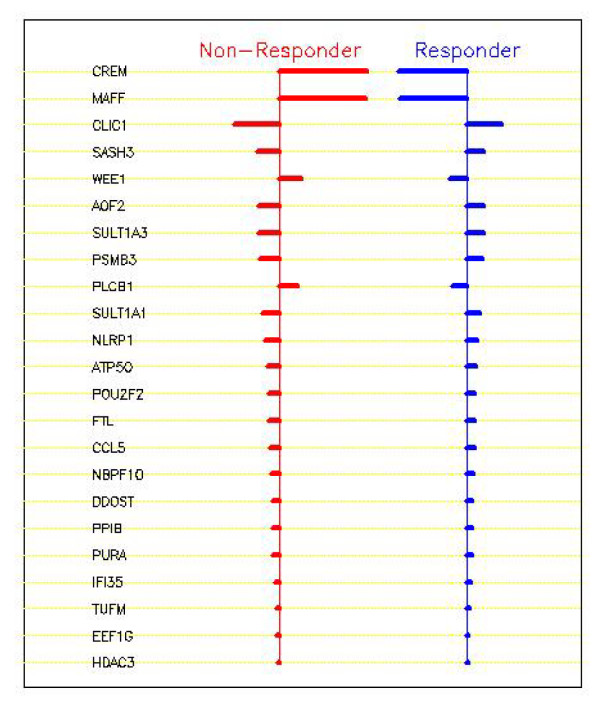
**Centroids for each gene in the classifier**. Centroid expression of the 23 genes in the genomic classifier for non-responders (red) and responders (blue). Lines to the left indicate relative increased levels of expression; lines to the right indicate relative decreased levels of expression. Some genes, for example the top two CREM and MAFF, are completely opposite in their expression in these two groups (down in non-responders, up in responders).

With this threshold 23 genes were selected to form the classifier that predict response to alefacept using these pre-treatment blood measurements (Table 2). Figure 3 shows the centroids of each gene in both responders and non-responder groups. A more stringent cut-off (threshold of 2.3) gave fewer genes (no. of genes = 19). However, while some genes appear to separate more clearly, we would like to use all 23 genes in a future prediction trial, as it makes it less likely to overlook genes that could be important.

**Table 2:** Genes that act as a genomic classifier for response to treatment of psoriasis with Alefacept.

Probe	Symbol	Description	Gene	av-rank-in- CV	prop- selected-in-CV
32067_at	CREM	cAMP responsive element modulator	1390	2.71	1.00

36711_at	MAFF	v-maf musculoaponeurotic fibrosarcoma oncogene homolog F (avian)	23764	2.86	1.00

36131_at	CLIC1	chloride intracellular channel 1	1192	4.71	1.00

40296_at	SASH3	SAM and SH3 domain containing 3	54440	11.71	0.86

36909_at	WEE1	WEE1 homolog (S. pombe)	7465	11.29	0.86

41122_at	AOF2	amine oxidase (flavin containing) domain 2	23028	14.86	1.00

32317_s_at	SULT1A3	sulfotransferase family, cytosolic, 1A, phenol-preferring, member 3	6818	14.14	0.71

1309_at	PSMB3	proteasome (prosome, macropain) subunit, beta type, 3	5691	15.14	0.57

35980_at	PLCB1	phospholipase C, beta 1 (phosphoinositide-specific)	23236	18.71	0.71

31804_f_at	SULT1A1	sulfotransferase family, cytosolic, 1A, phenol-preferring, member 1	6817	17.29	0.71

37127_at	NLRP1	NLR family, pyrin domain containing 1	22861	16.86	0.86

37029_at	ATP5O	ATP synthase, H+ transporting, mitochondrial F1 complex, O subunit (oligomycin sensitivity conferring protein)	539	18.86	0.57

37417_at	POU2F2	POU class 2 homeobox 2	5452	20.86	0.71

35083_at	FTL	ferritin, light polypeptide	2512	20.86	0.57

1404_r_at	CCL5	chemokine (C-C motif) ligand 5	6352	25.43	0.57

34279_at	NBPF10	neuroblastoma breakpoint family, member 10	440673	35.14	0.43

38791_at	DDOST	dolichyl-diphosphooligosaccharide-protein glycosyltransferase	1650	24	0.57

35823_at	PPIB	peptidylprolyl isomerase B (cyclophilin B)	5479	23.71	0.86

41344_s_at	PURA	purine-rich element binding protein A	5813	26.29	0.71

464_s_at	IFI35	interferon-induced protein 35	3430	34	0.57

39867_at	TUFM	Tu translation elongation factor, mitochondrial	7284	27.43	0.43

1676_s_at	EEF1G	eukaryotic translation elongation factor 1 gamma	1937	27.71	0.71

35821_at	HDAC3	histone deacetylase 3	8841	29.57	0.43

It is important to acknowledge the limitations of our study. The data-base for this study was small, as we were restricted to the clinical trial sample size, and the number of patients with good quality RNA and chip data. Although this data has been previously analyzed to find genes that were differentially expressed between responders and non-responders, this time we asked a different question of the data, specifically, could genes expressed in PBMCs before treatment *predict *response to alefacept. While we note that two of the non-responding patients were misclassified as responders, our preliminary conclusions suggest that this is a promising approach.

The genes in this classifier should be considered as a group. However some of the individual genes are of great interest. For example, cAMP response element modulator (CREM) is a gene that is highly increased in responders compared to non-responders, and this encodes activators and antagonists of camp-inducible transcription by differential splicing [17]. In systemic lupus erythematosis, phosphorylated CREM correlated with decreased production of IL-2 and anergy in T cells [18]. The pattern of expression of v-MAF avian musculoaponeurotic fibrosarcoma oncogene family (MAFF) is similar to CREF. This protein interacts with the upstream promoter region of the oxytocin receptor gene, and may be involved in the cellular stress response [19]. Chloride intracellular channel protein 1 (CLIC1, also called NCC27) has the opposite expression pattern with an increase in non-responders, and a decrease in responders. Although the role of this gene in inflammation is not entirely clear, this gene does function as a nuclear chloride channel protein.

NLR family, pyrin domain-containing 1 (NLRP1) is involved in activation of caspase-1 and caspase-5 as part of the NALP1 inflammasome complex. The formation of this complex is important in the processing and release of bioactive IL-1β and IL-18 [20]. NLRP1 is also involved in apoptosis. CCL5 (chemokine, cc motif, ligand 5, also called regulated upon activation, normally T expressed, and presumably secreted/RANTES) is a chemo-attractant for circulating monocytes, memory T helper cells, and eosinophils [21]. Thus there were several interesting genes in this list, although the list should be taken as a whole for its use as a genomic classifier.

## Conclusion

We conducted an alternative analysis of our previously published baseline peripheral blood microarray data [6], in order to determine the genes that would predict response to alefacept. We used a discriminant analysis method that performs sample classification from gene expression data [7]. The database for this study was small, limited by the sample size of the clinical trial, and makes our conclusions preliminary. This approach and data are presented to show how pre-treatment peripheral blood microarray data can be used to identify a novel set of genes and develop a "genomic classifier". This genomic classifier could predict response to treatment and thus help physicians in selecting psoriasis patients who could benefit from treatment with Alefacept. This genomic classifier now needs to be tested prospectively.

## Abbreviations

Abbreviations used in this article are PASI: Psoriasis Area and Severity Index; PBMCs: Peripheral blood mononuclear cells; FDR: False discovery rate; CREM: cAMP response element modulator; IL-2: Interleukin 2; v-MAF: Avian musculoaponeurotic fibrosarcoma oncogene family; CLIC1: Chloride intracellular channel protein 1; NLRP1: NLR family, pyrin domain-containing 1; CCL5: Chemokine, CC motif, ligand 5; MIG: monokine induced by *IFNγ*; STAT-1: signal transducer and activator of transcription 1; iNOS: inducible NO synthase.

## Competing interests

The authors declare that they have no competing interests.

## Authors' contributions

MSF performed data analysis, interpretation of results, and wrote the paper; KRS interpreted the results and wrote the paper; ASH performed data analysis; JGK conducted the clinical trial and performed data analysis and interpreted the results; and MAL performed data analysis, interpretation of results, and wrote the paper. All authors read and approved the final manuscript.

## Pre-publication history

The pre-publication history for this paper can be accessed here:

http://www.biomedcentral.com/1471-5945/10/1/prepub
